# Extension of Regression Discontinuity Designs (RDD) to evaluate time effects – A quasi-experimental approach using the data from the new form of care “Integrated cross-sectoral psycho-oncology (nFC- isPO)”

**DOI:** 10.1371/journal.pone.0343414

**Published:** 2026-02-26

**Authors:** Anna Hagemeier, Kerstin Daniela Rosenberger, Martin Hellmich

**Affiliations:** 1 Institute of Medical Statistics and Computational Biology, Medical Faculty, University of Cologne, University Hospital Cologne, Cologne, Germany; 2 Department of Medical Statistics, University Medical Center Göttingen, Göttingen, Germany; Deccan School of Pharmacy, INDIA

## Abstract

**Objective:**

Randomised controlled trials (RCTs) are considered the gold standard in clinical research. However, randomization isn’t always feasible, e.g., due to ethical considerations. As a result, quasi-experimental designs, such as the regression-discontinuity design (RDD), have been advocated as valuable alternatives and are increasingly employed in health sciences. So far, however, there is no way to take repeated measures into account in an RDD analysis, although repeated measures are not uncommon in medical research. This article proposes an extension of the RDD methodology to incorporate repeated measures within the statistical analysis.

**Methods:**

The article introduces consistent mathematical notation for combining a univariate RDD with a linear mixed model (LMM) to account for repeated measures. The application is presented using data from the nFC-isPO (N = 1,417), where the Hospital Anxiety and Depression Scale (HADS) was employed to assess anxiety and depression in newly diagnosed cancer patients over a 12-month treatment period. The HADS scores were measured at baseline (T1), after 4 months (T2) and after 12 months (T3). Patients were assigned to control (psychosocial care) or treatment (psycho-oncological-psychotherapeutic care) group based on a predetermined HADS threshold at T1.

**Results:**

The average treatment effect (ATE) was similar in both univariate RDD and LMM-RDD when applied to complete cases. Including all available data (T2 and/or T3), univariate RDD analyses were based on different samples for each time point. At T2, the ATE sign reversed from negative to positive, suggesting a change in discontinuity direction. At T3, the ATE magnitude nearly doubled compared to the complete case analysis. LMM-RDD estimated treatment effects were higher than those from univariate RDD. Nevertheless, none reached statistical significance. The time effect (Δt), representing the difference in treatment effects between two time points (complete case: Δt=0.577; T2 and/or T3 available: Δt=0.491), was not significant (p = 0.855; 95%-CI: [−5.589; 6.743]; p = 0.869, 95%-CI: [−5.328; 6.310]).

**Conclusion:**

Extending the RDD to incorporate repeated measures within a linear mixed model (LMM-RDD) provides a more robust and comprehensive analytical approach when an RCT is not feasible. This approach not only addresses the challenges of missing data and an unbalanced number of outcome measures, but also allows for the identification of time-varying treatment effects that may not be discernible using traditional RDD.

**Trial registration:**

The study was registered in the German Clinical Trials Registry on 30 October 2018 under the ID “DRKS00015326”.

## 1. Introduction

Although the randomised controlled trial (RCT) is the gold standard in clinical research, it cannot be done in every setting. For example, if the intervention to be evaluated has already been implemented in standard practice withholding from certain patients may not be an ethical option anymore [1]. However, a quasi-experimental design, mimicking randomization of individuals, might still provide valid information to evaluate the intervention. Specifically, in the regression discontinuity design (RDD), which in its simplest form is based on ordinary linear regression, a predefined threshold is used to assign cases to treatment groups. The control group receives standard care, while the intervention group receives standard care plus extended care. This assignment is based on a continuous parameter measured before the intervention. A quasi-randomisation is created around the threshold within a certain pre-defined range (bandwidth). Within this range, the treatment effect can be estimated, visible as the discontinuity of two regression lines at the threshold [2]. The design has its origins in 1960 [3], however was rarely used in the health sciences until 2010, and thereafter has become increasingly popular, especially in the last five years [4]. Despite the increasing use of this design and the ability to examine causal relationships without random assignment, there is currently no way to account for repeated measures within the RDD. Repeated measures are common in medical research and many studies use them to estimate treatment effects over time [5], including the new Form of Care and integrated cross-sectoral Psycho-Oncological (nFC-isPO) study. The nFC-isPO ran from 2017 to 2022 and aimed to reduce anxiety and depression in newly diagnosed cancer patients during 12 months of care. The Hospital Anxiety and Depression Scale (HADS) was used to measure the patient’s burden at time of diagnosis (baseline, T1), after 4 months (T2) and after 12 months of treatment (primary outcome, T3) [6,7]. The study was conducted and analysed using the RDD where both the continuous assignment variable and the outcome variable are HADS scores measured at different time points. The assignment into control (HADS≤14) and intervention (HADS≥15) group was done using the baseline HADS value (T1, assignment variable) with c = 14.5 as the cut-off value [8,9]. This threshold value is established in psycho-oncological care and was used in the isPO study due to its clinical relevance for the classification of ‘moderate to high psychological stress’ [7,10,11]. For final evaluation of the study, the time points T2 and T3, were analysed separately, as with the standard RDD approach it is not possible to take into account time effects. In order to obtain additional information on temporal relationships, mixed models for repeated measures were applied in secondary analyses [12]. However, an integrated approach considering time effects within the RDD analysis was not yet described in literature.

The aim of this article is to oppose and formally describe mixed model repeated measures approach to analyse the RDD for a scenario with continuous outcome measured at 3 time points (baseline T1, T2, T3) and to exemplify this novel approach with recent data from the nFC-isPO.

## 2. Methods

### 2.1 Model extension

To address the effect over time, the functional form of the RDD, which is based on a simple linear regression, needs to be extended to a linear mixed model for repeated measures.

Defining Y as the continuous outcome for n observations, X as the covariate that is the continuous assignment variable for each observation and c as the cut-off value for treatment assignment based on the covariate X. Furthermore, define $x_{i}^{*}=x_{i}-c$ as the measure of the distance to the cut-off and let 1 denote the indicator function for the treatment allocation, that equals $\text{0}\;if\;x_{i}\;\leq \;c$ (control group) or $\text{1}\;if\;x_{i}>c$ (treatment) (the so-called “sharp RDD”).

**Fig 1:**
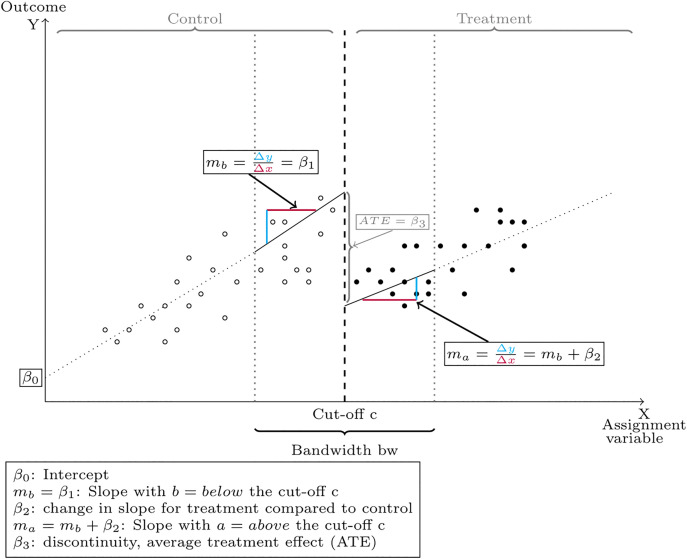
Plot of univariate Regression-Discontinuity-Design.

Then the univariate (i.e., considering only one time point) RDD equation [2] is given as


$$\begin{array}{c} y_{i}\;=\;\beta_{0}\;+\;\beta_{1}{x_{i}}^{*}\;+\;\beta_{2}1\left(x_{i}\;>\;c\right){x_{i}}^{*}\;+\;\beta_{3}1\left(x_{i}>c\right)\;+\;e_{i} \end{array}$$

$$=\;\beta_{0}\;+\;\beta_{1}{x_{i}}^{*}+\left\{ \begin{array}{c} \;\;{\;\;\beta }_{2}{x_{i}}^{*}\;+\;\beta_{3}\;+\;e_{i}\;,\;if\;x_{i}\text{ > }c\\ \;\;\;\;e_{i}\;,\;if\;x_{i}\;\leq c \end{array}\right.\;\;$$ (1)

with i=1,2,…,n patients and residuals $e_{i}\sim N\left(\text{0},\sigma^{\text{2}}\right)$ with mean of 0 and variance of σ² as the random error. Equation (1) corresponds to Fig 1 where $\beta_{\text{0}}$ is the intercept, $\beta_{\text{1}}$ denotes the slope $\left(m_{b}\right)$ of the regression line below the cut-off (control), $\beta_{\text{2}}$ denotes the change in slope ($m_{a}=m_{b}+\beta_{\text{2}})$ for the regression line above the threshold (treatment) compared to the control group and $\beta_{\text{3}}$ denotes the average treatment effect (ATE, discontinuity/ “jump” at cut-off).

A more detailed description of the RDD can be found in [2]. Equation (1) is then extended to a linear mixed model (LMM) for repeated measures over time:


$$\begin{array}{c} y_{i,j}=\beta_{\text{0}}+\beta_{\text{1}}{x_{i}}^{*}+\beta_{\text{2}}1\left(x_{i}>c\right){x_{i}}^{*}+\beta_{\text{3}}1\left(x_{i}>c\right)+{S_{i,j}+b}_{i\text{0}}+e_{i,j} \end{array}$$ (2)

$$\begin{array}{c} with\;S_{i,j}=\sum_{j^{\prime }=\text{2}}^{J}{D_{j^{\prime },j}[\beta }_{\text{4},j^{\prime }}+\beta_{\text{5},j^{\prime }}x_{i}^{*}+\beta_{\text{6},j^{\prime }}{1\left(x_{i}>c\right)x}_{i}^{*}+\beta_{\text{7},j^{\prime }}1\left(x_{i}>c\right)]\; \end{array}$$

where Y is defined as the continuous outcome for patient i=1, 2, …, n  and time points j=1,2,3,…, J.

$D_{j^{\prime },j}=\left\{ \begin{array}{c} \;\;\;\;\text{1},\;if\;j^{\prime }=j\\ \;\;\;\;\text{0},\;otherwise\; \end{array}\right.\;$ is a dummy variable to model the time effect with running index $j^{\prime }=\text{2},\text{3},\ldots ,J$.

$\beta_{\text{4},j^{\prime }}$ denotes the change in intercept at time point $j^{\prime }$ compared to time point 1. $\beta_{\text{5},j^{\prime }}\;and\;\beta_{\text{6},j^{\prime }}$ denote the changes in slopes at time point $j^{\prime }$ compared to time point 1 and $\beta_{\text{7},j^{\prime }}$ is the change in the treatment effect for time point $j^{\prime }$ compared to time point 1. $b_{\text{i}\text{0}}\sim N\left(\text{0},\sigma_{b}\right)$, with a mean of 0 and a variance $\sigma_{b}$, is the random intercept for patient i to account for stochastic dependence over time (repeated measures) and $e_{i,j}\sim N(\text{0},\sigma )$ with a mean of 0 and a variance σ denotes the vector of error terms for patient i at time point j. A random slope could be defined in a similar way for each coefficient, but is not considered further below for the purpose of simplicity.

For illustration, equation (3) shows the example for just two outcome measures, i.e., two time points with J=2 (j=1,2 and $j^{\prime }=\text{2}$) for i=1,2,…, n patients:


$$\begin{array}{c} y_{i,j}=\beta_{\text{0}}+\beta_{\text{1}}{x_{i}}^{*}+\beta_{\text{2}}1\left(x_{i}>c\right){x_{i}}^{*}+\beta_{\text{3}}1\left(x_{i}>c\right)+D_{j^{\prime },j}[\beta_{\text{4},j^{\prime }}+\beta_{\text{5},j^{\prime }}x_{i}^{*}+\beta_{\text{6},j^{\prime }}{1\left(x_{i}>c\right)x}_{i}^{*}+\beta_{\text{7},j^{\prime }}1\left(x_{i}>c\right)]+b_{\text{0}i}+e_{i,j} \end{array}$$

$$\begin{array}{c} =\beta_{\text{0}}+\beta_{\text{1}}{x_{i}}^{*}+\left\{ \begin{array}{c} \;\;\;\;\beta_{\text{2}}x_{i}^{*}+\beta_{\text{3}}+{D_{j^{\prime },j}[\beta }_{\text{4},j^{\prime }}+\beta_{\text{5},j^{\prime }}x_{i}^{*}+\beta_{\text{6},j^{\prime }}x_{i}^{*}+\beta_{\text{7},j^{\prime }}]+b_{\text{0}i}+e_{i,j},\;if\;x_{i}>c\\ \;\;\;\;{D_{j^{\prime },j}[\beta }_{\text{4},j^{\prime }}+\beta_{\text{5},j^{\prime }}x_{i}^{*}]+\;b_{\text{0}i}+e_{i,j}\;,\;if\;x_{i}\leq c \end{array}\right.\; \end{array}$$ (3)

**Fig 2:**
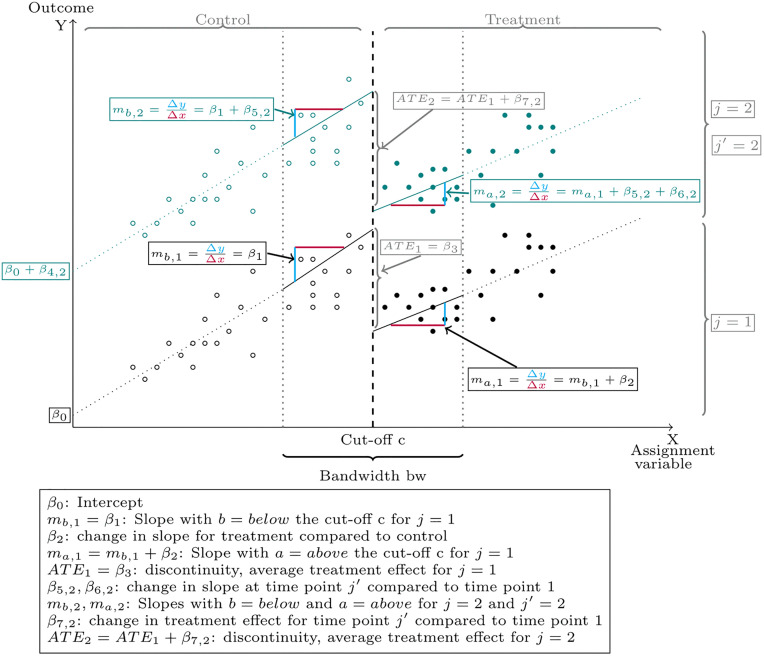
Plot of the LMM-RDD for the special case of j = 1,2.

With $D_{\text{2},\text{1}}=\text{0}$ and $D_{\text{2},\text{2}}=\text{1}$ a schematic representation of equation (3) is given in Fig 2.

The extended model now considers the correlation between repeated measures for each observation. Moreover, allows the inclusion of cases with only one follow-up outcome measure, assuming that these are missing at random. The extension of the RDD equation to a linear mixed model RDD for repeated measures allows the evaluation of the time effect within an RDD analysis. This is achieved by estimating the difference between two regression coefficients corresponding to the treatment effects at individual time points.

### 2.2 Calculation of the time effect

To evaluate the time effect, the standard error, the corresponding confidence interval as well as the corresponding t-statistic- and p-value can be calculated for the difference between two regression coefficients [13,14]. First, the difference between the coefficients of interest is calculated, where these coefficients represent the treatment effect at different time points j,k∈{1,2,…,J}. The difference corresponds to the time effect and is given by $\Delta t={\hat{ATE}}_{j}-{\hat{ATE}}_{k}$ with j≠k and where the head notation denotes an estimate. The corresponding standard error can be derived using Bienaymé’s theorem [15]:


$$SE\left(\Delta t\right)=\;\sqrt{Var\left(\Delta t\right)}=\sqrt{Var\left({\hat{ATE}}_{j}-{\hat{ATE}}_{k}\right)}=\;\sqrt{Var\left({\hat{ATE}}_{j}\right)+Var\left({\hat{ATE}}_{k}\right)-2\cdot Cov\left({\hat{ATE}}_{j}\;,\;{\hat{ATE}}_{k}\right)}$$ (4)

The standard error and the estimated difference of the coefficients (time effect) can then be used to calculate the corresponding t-value: $t_{\Delta \text{t}}=\frac{\Delta t}{SE(\Delta t)}$ and via the z-score (which approximates the t-value for large samples [16]) the p-value can be calculated by $p_{\Delta t}=\text{2}\cdot \left(\text{1}-P\left(Z\leq |z|\right)\right)$. Finally, a confidence interval (CI) for the difference between the coefficients (time effect) can be determined:


$$\text{9}\text{5}\%\;CI=\Delta t\pm z_{\frac{\alpha }{\text{2}}}SE(\Delta t)\text{ with }z_{\frac{\alpha }{\text{2}}}=\text{1}.\text{9}\text{6}.$$

In the following, both the simple linear RDD and the LMM-RDD for repeated measures (with unstructured variance-covariance matrix over time) are exemplified using the data from the isPO study. All analyses are exploratory and were done using R 4.4.0 [17]. Results were considered statistically significant at a significance level of α = 0.05 when p ≤ 0.05.

### 2.3 Data collection and analysis population

**Fig 3:**
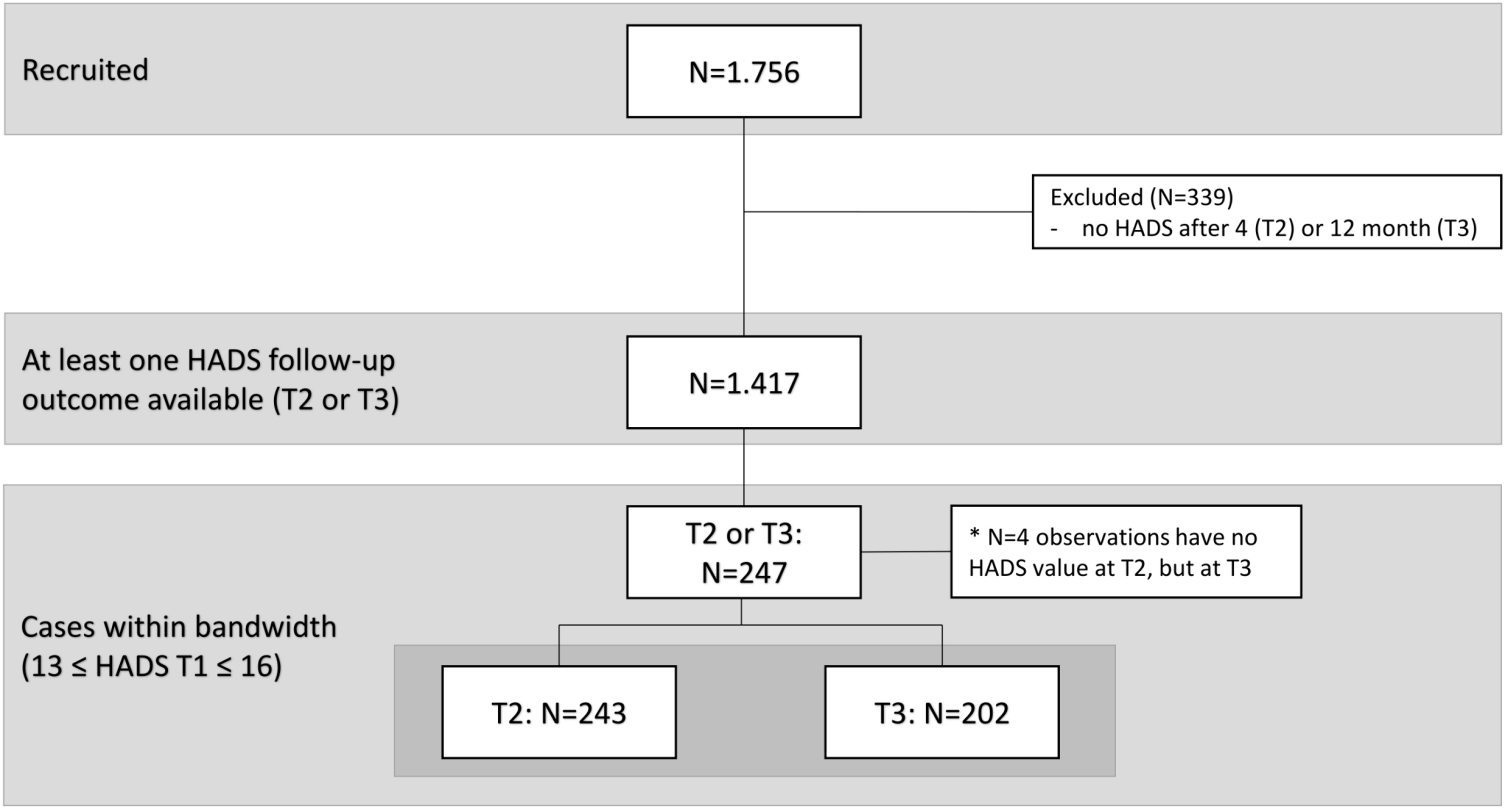
Flowchart of study and analysis population.

Between January 15, 2019 and March 31, 2021, N = 1,756 newly diagnosed cancer patients were enrolled in four isPO care networks (Cologne, Troisdorf, Mönchengladbach and Neuss) in North Rhine-Westphalia (NRW) and documented in the CAPSYS^2020^ computer assistance system developed specifically for the project. Each patient received individualized care and was therefore assigned to different treatment groups based on their needs as determined by their HADS scores (baseline, T1) at study enrolment. Patients with HADS scores ≤ 14 received a psychosocial intervention (control group) and patients with scores ≥ 15 received psychooncological, psychotherapeutical treatment (intervention group). After 4 (T2) and 12 months (T3), the HADS was measured again, and the treatment effect for the primary analysis in isPO was analysed separately for the two measurement time points (T2, T3) using standard RDD within a predefined bandwidth (HADS between 13–16) around the cut-off value [9]. Since a HADS result had to be available at least after 4 or 12 months for evaluation, 339 cases were excluded. This results in an N of 1,417 cases for the analysis population to estimate the treatment and time effects via LMM-RDD (Fig 3). All patients gave their written informed consent.

### 2.4 Ethics statement

The study was conducted in accordance with the Declaration of Helsinki and the Ethics Committee of the Medical Faculty of the University of Cologne has approved the isPO project (18–092). The isPO study is registered in the German Clinical Trials Registry (DRKS00015326). All participants have been informed about the aim of the study and about the voluntary nature of participation and have signed a written informed consent form for participation in the isPO care programme and data processing.

## 3. Results

### 3.1 Characteristics of the patients

N = 1,417 nFC-isPO patients had at least one HADS follow-up outcome available at 4 (T2) or 12 months (T3). Limited to the relevant bandwidth of 13–16 points on the HADS scale at baseline (T1), N = 247 cases remain for primary analysis. Around two thirds of them were female. 61.5% reported to live in a relationship and in the same household with their respective partners. Detailed information about main characteristics of the patients can be found in Table 1.

**Table 1:** Sociodemographic and clinical patient characteristics for analysis population and the population within the bandwidth (HADS between 13 to 16) for the primary analysis of the RDD.

	Bandwidth (N = 247)	Total (N = 1,417)
**Gender**		
Female	160 (64.8%)	902 (63.7%)
Male	87 (35.2%)	515 (36.3%)
**Age**		
Mean ± SD	56.1 ± 14.0	57.1 ± 13.0
Median [Q1; Q3]	57.0 [49.0;66.5]	58.0 [50.0;66.0]
**Care level**		
Cancer self-help (care level 1)	7 (2.8%)	122 (8.6%)
Psychosocial cancer counselling (care level 2)	109 (44.1%)	523 (36.9%)
Psycho-oncological psychotherapy (care level 3)	131 (53.0%)	772 (54.5%)
**Relationship status**		
No relationship	68 (27.5%)	331 (23.4%)
In relationship. shared household	152 (61.5%)	942 (66.5%)
In relationship. separate households	16 (6.5%)	90 (6.4%)
Missing	11 (4.5%)	54 (3.8%)
**ISCED**		
Primary education	3 (1.2%)	23 (1.6%)
Lower secondary education	11 (4.5%)	90 (6.4%)
Upper secondary education	169 (68.4%)	956 (67.5%)
Bachelor or equivalent	10 (4.0%)	94 (6.6%)
Master or equivalent	43 (17.4%)	199 (14.0%)
Doctoral or equivalent	4 (1.6%)	17 (1.2%)
Missing	7 (2.8%)	38 (2.7%)
**Severe disability**		
No	168 (68.0%)	959 (67.7%)
Yes	74 (30.0%)	432 (30.5%)
Missing	5 (2.0%)	26 (1.8%)
**Chronic illness**		
No	140 (56.7%)	815 (57.5%)
Yes	102 (41.3%)	576 (40.6%)
Missing	5 (2.0%)	26 (1.8%)
**ICD 10 / Tumor-entities**		
Digestive organs (C15-C26)	31 (12.6%)	219 (15.5%)
Respiratory and intrathoracic organs (C30-C39)	18 (7.3%)	135 (9.5%)
Skin (C43-C44)	14 (5.7%)	82 (5.8%)
Breast (C50)	72 (29.1%)	373 (26.3%)
Female genital organs (C51-C58)	13 (5.3%)	83 (5.9%)
Lymphoid, hematopoietic and related tissue (C81-C96)	26 (10.5%)	147 (10.4%)
Other (each<5%)	67 (27.1%)	345 (24.3%)
Missing	6 (2.4%)	33 (2.3%)
**Death**		
No	236 (95.5%)	1,360 (96.0%)
Yes	11 (4.5%)	57 (4.0%)

### 3.2 Results of the univariate and mixed model RDD

Table 2 shows the results of the univariate and linear mixed model RDD for the cases with complete follow-up data and for the case where either T2 and/or T3 are available. To illustrate the difference in results, depending on the data used, both modelling approaches were applied to cases with complete follow-up data to show that the result, the $\hat{ATE}$, is the same (${\hat{ATE}}_{T\text{2}}=-\text{0}.\text{213}$; ${\hat{ATE}}_{T\text{3}}=\text{0}.\text{364}$) regardless of whether the RDD is performed in its usual univariate or extended linear mixed model form. When the models are applied to patients with at least one follow-up visit (T2 and/or T3 available), univariate RDD estimates are based on (slightly) different samples of cases for each time point. In the univariate case for T2, the sign changes from negative to positive and the discontinuity changes direction. Although the sign of the $\hat{ATE}$ estimate changes, both estimates were very close to zero with wide confidence intervals. For T3, the $\hat{ATE}=\text{0}.\text{676 }$ is almost twice as high as in the model applied to the cases with complete follow-up data, but again the estimate is very small with a wide confidence interval. The treatment effects estimated by the LMM-RDD are even higher than the effects calculated in the univariate models, but still with large confidence intervals. None of the results reached statistical significance.

**Table 2:** Results of the univariate and the linear mixed model RDD.

	T2						T3					
	N_bw_	ATE	Std. Err.	t-value	p-value	95% CI	N_bw_	ATE	Std. Err.	t-value	p-value	95% CI
**Complete case**												
Univ. RDD	198	−0.213	1.759	−0.121	0.904	[-3.682; 3.255]	198	0.364	2.070	0.176	0.861	[-3.719; 4.447]
LMM-RDD	198	−0.213	1.921	−0.111	0.912	[-3.994; 3.567]	198	0.364	1.921	0.189	0.850	[-3.401; 4.129]
**T2 or T3 not missing**												
Univ. RDD	243	0.322	1.665	0.193	0.847	[-2.958; 3.601]	202	0.676	2.053	0.329	0.742	[-3.372; 4.724]
LMM-RDD	243	0.481	1.775	0.271	0.786	[-3.01; 3.973]	202	0.972	1.870	0.520	0.603	[-2.693; 4.637]

### 3.3 Effect over time

**Fig 4:**
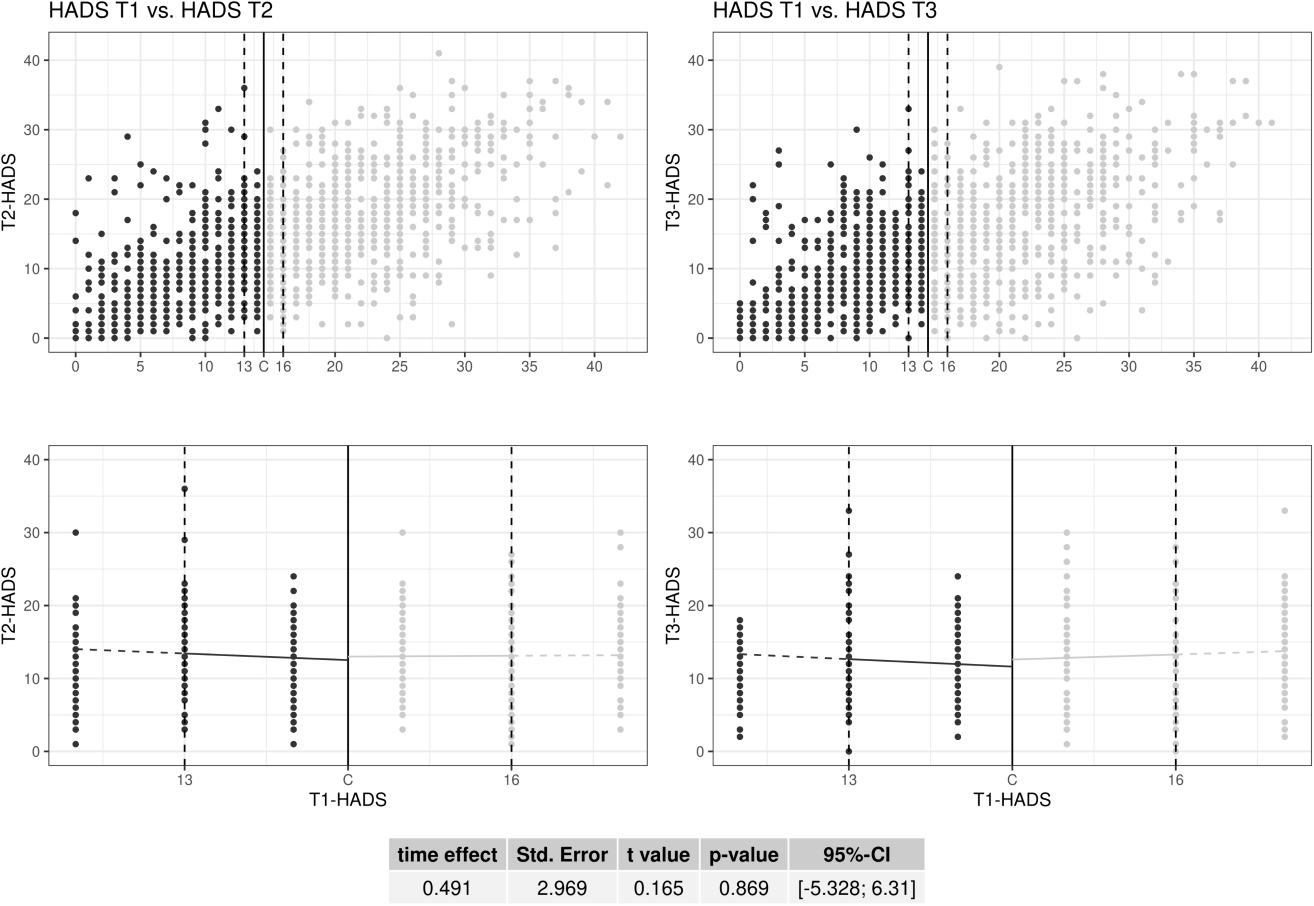
Scatter plot (four panels) of the linear mixed model regression discontinuity design and the estimated time effect (Δt) for the comparison of the patients’ outcome HADS scores at T2 (after 4 months) and T3 (after 12 months of treatment).

Within the mixed model, it is now possible to calculate the time effect as the difference between the two treatment effects at the different time levels. The time effect with cases with complete follow-up data was Δt=0.364−(−0.213)=0.577 (p=0.855; 95%−CI: [−5.589; 6.743] with a standard error of 3.146 and a t-value of 0.183. Fig 4 is the graphical representation of the LMM-RDD in which the HADS value of T2 or T3 are not missing (see last row of Table 2). In order to obtain an overview of the data, the upper scatter plots show the relationship between the HADS values at time T1 and the respective outcome measurements at T2 and T3 over the entire value range of the HADS, whereby it can be seen that the respective outcome value increases with increasing initial value. Below is the representation of the LMM-RDD within the specified bandwidth with an $\hat{ATE}$ of 0.481 for T2 and an $\hat{ATE}$ of 0.972 for T3 (both not significant; s. Table 2). The table below all four plots contains the estimated time effect (Δt=0.491), which is also not significant (p=0.869, 95%−CI: [−5.328; 6.310]).

## 4. Discussion

The RDD gives a promising alternative for drawing causal conclusions in case random group assignment is not feasible. Previously, it was not possibility to include repeated measures in the RDD. In order to close this gap, the simple linear RDD was extended to a repeated measure linear mixed model RDD by including the factor time and a random intercept (see equation 2). To demonstrated the advantages and differences of the linear mixed model RDD compared to the univariate RDD, data from the nFC-isPO study were reanalysed. First, only cases with complete follow-up data and, second all data (T2 and/or T3 are available) were considered. With the univariate method, a direct comparison between the time points on the basis of the calculated effects for cases with complete follow-up data is not possible. A valid 95% CI needs to account for the statistical dependence of both time points (repeated measures). Therefore, only a graphical interpretation or comparison of the RDD lines is possible. When using all available data, a graphical analysis is still possible, but even more limited, as the calculations of the treatment effect are based on (slightly) different samples. This implies that the database should be limited to cases with complete follow-up data or cases with valid primary endpoint, thus information is deliberately excluded from the analysis. With a linear mixed model a restriction to complete follow-up data is not required. When effects are estimated, particularly due to treatment and time, the correlation structure of repeated measures is accounted for [12].

### 4.1 Limitations and open issues

A major limitation of conducting a study in the medical field is the often limited sample size, which poses a problem when using an RDD. Simulation studies have shown that RDDs require a larger amount of data than RCTs in order to detect (moderate) treatment effect sizes and achieve comparable statistical power. The reason for this is that only units that are close to the threshold value can be compared – i.e., in the pre-specified range where quasi-randomisation can be assumed [18–20]. This might be one explanation for the overall insignificant results, including the rather wide confidence intervals of the analyses performed. Moreover, with regard to the application of the methods to the nFC-isPO study data, there was the limitation that the cases within the bandwidth were either too similar in terms of the individual need for psycho-oncological care or the burden above the defined threshold was too low to be able to demonstrate a relevant treatment effect.

This article extends a current method and demonstrates its feasibility with empirical data. For clarity, the analysis was limited to a random intercept model; however, further research could incorporate random slopes to gain valuable insights into individual differences over time. Further investigation is also needed to systematically compare the performance of the univariate and LMM-RDD approaches in a simulation study, considering various sample sizes, effect sizes, and missing data patterns.

## 5. Conclusion

Applying a linear mixed model (LMM) to a regression discontinuity design (RDD) with repeated measures is a powerful approach to analyse longitudinal data in case a RCT cannot be conducted. The presented combined model (LMM-RDD) enables the estimation of time effects, which has not been possible with the existing approaches, yet.

## Abbrevations:

ATE: average treatment effect
CAPSYS^2020^: computer-assisted assistance system in isPO
HADS: Hospital Anxiety and Depression Scale
LMM-RDD: linear mixed model RDD
nFC-isPO: new form of care integrated, cross-sectoral psycho-oncology
NRW: North Rhine-Westphalia
RCT: Randomised controlled trial
RDD: Regression discontinuity design
